# Supplementary material for: Creep mechanical tests and shear rheological model of the anchorage rock mass under water‒rock coupling
Source: PLoS One. 2023 Apr 14;18(4):e0284569. doi: 10.1371/journal.pone.0284569 (PMC10104343; doi:10.1371/journal.pone.0284569)
Supplement: Attachment — Submitted filename: Response to Reviewer Comments.doc [file pone.0284569.s002.doc]

Response to the comments of manuscript RMRE-D-22-00429R1

We thank the editor and reviewers for the time and effort that they put in to carefully review our manuscript. The comments were very helpful for improving the manuscript. We have carefully modified the corresponding contents, which are in red font in the revised manuscript.

The responses to each comment are listed as follows:

**Associate Editor:**

Thank you for submitting your manuscript to PLOS ONE. After careful consideration, we feel that it has merit but does not fully meet PLOS ONE’s publication criteria as it currently stands. Therefore, we invite you to submit a revised version of the manuscript that addresses the points raised during the review process.

Reply: Thank you for the suggestions. We have revised the paper based on each comment.

# Reviewer #2:

# (1). Authors gave the results of uniaxial compression tests and direct shear tests as shown in the Response to the comments, but they are not found in the manuscript, please add them in the manuscript.

Reply: Thank you for the suggestions. We have supplemented the figure in the revised manuscript as follows:

The relevant test figures are shown in Fig. 1-2.

**Fig. 1** Uniaxial stress-strain curve

**Fig. 2** Peak shear stress curve under normal stress

# (2). For the problem of 16 that I stated before: “Can this shear rheological model describe the steady creep of the anchorage rock mass under water‒rock coupling? If it can, please demonstrate it.” The response just shows a figure, and it has no a word to describe it. As well as, this content should be incorporated into the manuscript.

Reply: Thank you for the suggestions. The rheological model can describe the steady-state creep of the anchorage rock mass under water-rock coupling. We have supplemented the figure and description in the revised manuscript as follows:

**Fig. 3** Steady-state phase fitting curve

By connecting the nonlinear rheological element with the coupled model of anchoring rock mass in series, the coupled model of water‒rock under water cut conditions can be obtained, which can be used to study and analyse the steady creep of the anchorage rock mass under different water contents. The fitting curve of the stable stage is shown in Figure 14. It can be seen from the figure that the coupled model can well reflect the change law of the anchorage rock mass in the stable stage. The proposed model provides a theoretical basis for the study of anchored rock masses under water cut conditions and new methods for the stability and reinforcement of rock masses.
